# Lineage-specific expansion of DNA-binding transcription factor families

**DOI:** 10.1016/j.tig.2010.06.004

**Published:** 2010-09

**Authors:** Varodom Charoensawan, Derek Wilson, Sarah A. Teichmann

**Affiliations:** MRC Laboratory of Molecular Biology, Cambridge CB2 0QH, UK

## Abstract

DNA-binding domains (DBDs) are essential components of sequence-specific transcription factors (TFs). We have investigated the distribution of all known DBDs in more than 500 completely sequenced genomes from the three major superkingdoms (Bacteria, Archaea and Eukaryota) and documented conserved and specific DBD occurrence in diverse taxonomic lineages. By combining DBD occurrence in different species with taxonomic information, we have developed an automatic method for inferring the origins of DBD families and their specific combinations with other protein families in TFs. We found only three out of 131 (2%) DBD families shared by the three superkingdoms.

## Phylogenetic analysis of DNA-binding transcription factor families

All sequence-specific transcription factors (TFs) contain DNA-binding domains (DBDs), evolutionary units that mediate the specificity of the TF–DNA interaction. Domain-based analysis of TFs is thus effective functionally as well as phylogenetically. TFs and their binding targets have been under intensive study, and previous key publications on TFs and DBDs tended to focus on specific phylogenetic groups [1–4]. Here, we analyze the distribution of all known DBDs in 538 organisms from superkingdoms Bacteria, Archaea and Eukaryota. TF and DBD classifications were obtained from the DBD database [5], a transcription factor resource that annotates TFs based on the presence of DBDs from a manually curated list. The DBD database predicts TFs in all publicly available genomes from diverse phylogenetic lineages using a single platform, and is thus an ideal resource for exploring the phylogenetic distribution of TF families across the tree of life. We provide an overview of conserved and lineage-specific DBD families, using 131 Pfam domains [6] classified as DBDs to illustrate our findings. Note that what we discuss here for Pfam DBDs applies also to 87 SCOP families [7] classified manually as DBDs by the DBD database (see the supplementary material online for a complete list of genomes and DBD families).

## TF DBD families are highly lineage-specific

**Figure 1:**
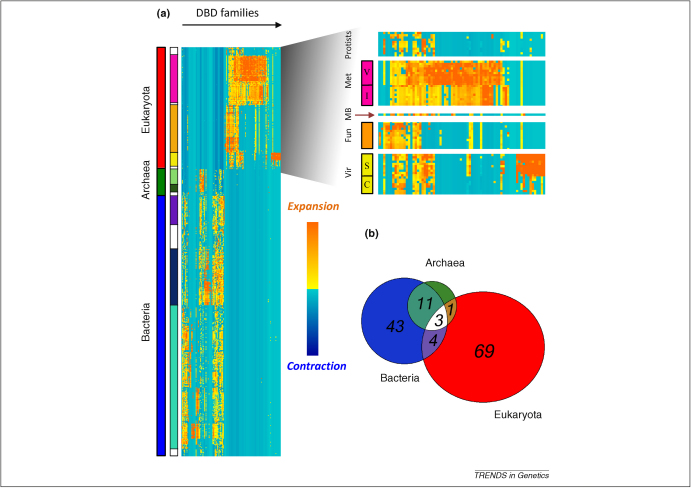
Lineage-specific expansion patterns of DBD families. **(a)** The heatmap demonstrates the specific expansion patterns of DBD families between eukaryotic and prokaryotic genomes. Columns correspond to DBD families hierarchically clustered by their occurrence patterns in different species. Rows represent species ordered using the NCBI taxonomy. Orange indicates relative DBD expansion and blue represents contraction. The vertical coloured bars to the left of the heatmap indicate superkingdoms, kingdoms, or phyla to which species belong. Eukaryota (red) is divided into three kingdoms: Metazoa (pink), Fungi (orange) and Viridiplantae (yellow). Euryarchaea and Crenarchaea are labelled in pale and dark green, respectively. Bacteria are labelled using shades of blue: Actinobacteria (purple), Firmicutes (navy) and Proteobacteria (pale blue). The white areas in the right-hand coloured bar are species that do not belong to the main kingdoms/phyla mentioned above, e.g. protists and choanoflagellate. Specific patterns of occurrence were observed within the eukaryotic species. At the right-hand side is shown the detailed expansion patterns of selected eukaryotic lineages: protists including Mycetozoa (*Dictyostelid*) and Stramenopiles, animals including V for vertebrates and I for invertebtrates, MB for *M. brevicollis* (choanoflagellate), fungi, and plants including S for streptophyta (land plants) and C for chlorophyta (green algae). **(b)** A Venn diagram representing the number of Pfam DBD families that have taxonomic limits belonging to the three main superkingdoms. Only 19 out of 131 (15%) DBDs were found in more than one superkingdom, whereas most of these DBDs are shared by Bacteria and Archaea but not by Eukaryota. Only three DBD families (CSD, HTH_psq, and HTH_3) are shared by all of the superkingdoms.

Earlier, we have introduced a heatmap representation to aid visualisation of the expansion and contraction of DBD families in order to investigate the distribution of DBDs in different lineages [5] (Figure 1a). Each column of the heatmap corresponds to a DBD family and each row represents a species. Species are ordered according to the NCBI taxonomic tree, an expertly curated taxonomic hierarchy [8]. The *Z*-score of a number of TFs containing a particular DBD family of interest was calculated for each family to highlight the organisms in which the family is expanded relative to other species. Orange indicates positive *Z*-scores and thus a relative expansion of the DBD in that particular lineage and blue indicates negative *Z*-scores or a contraction. The distinct expansion pattern of different groups of DBDs in prokaryotes and eukaryotes implies that the DBD families are highly specific to two types of cells: nucleate and anucleate.

In addition to the heatmap, we have developed a new simple method for inferring the origin of protein domains. By combining DBD family occurrence with taxonomic information from the NCBI taxonomy tree, we demonstrate that the method is able to estimate when each DBD family emerged. We term this the taxonomic limit. The same method is used to estimate when the combinations of DBDs and other protein families in TFs emerged. We provide the taxonomic conservation density, which is the fraction of species containing the DBD out of the total number of species within taxonomic clades (see Box 1 for an example of the calculation steps and see the supplementary material online for a complete list of taxonomic limits and conservation densities).

Using our method, we found that 19 out of 131 (15%) DBDs have cellular organisms as their taxonomic limits (shared by more than one superkingdom). Eleven of these DBDs are shared by Archaea and Bacteria but not Eukaryota, and only three (2%) are shared by all three superkingdoms (Figure 1b). When we apply the same method to all Pfam domains, we observed that 33% have cellular organisms as taxonomic limits, suggesting that the repertoires of DBD families are more lineage-specific than proteins with other functions. This conclusion is in line with the results of an earlier study that used a different method [9].

## Uniform expansion pattern of DBD families in prokaryotes

Focusing on the prokaryotic genomes, helix-turn-helix is by far the commonest DBD structure [1]. The majority of prokaryotic DBDs belong to the winged helix structural class, which might explain the uniform expansion of DBD occurrence observed here. Archaea are thought to be phylogenetically closer to Eukaryota and have more closely related core components of transcription machinery, such as RNA polymerases and basal TFs [1,4]. Interestingly, our heatmap and taxonomic limit assignments suggest a greater number of archaeal DBDs shared with Bacteria than with Eukaryota. Examples of DBDs shared by the two prokaryotic superkingdoms Archaea and Bacteria are Fe_dep_repress (iron-dependent repressor), MarR (antibiotic resistance) and NikR (nickel-responsive regulator). These DBD families regulate specific genes required for adaptation to environmental stress, and might have been established and maintained through multiple horizontal gene transfers [1,10].

The heatmap shown in Figure 1a suggests that the prokaryotic DBD distribution is widespread among the prokaryotic species and there is no clearly distinguishable expansion scheme within the three major bacterial phyla in our dataset: Actinobacteria, Firmicutes and Proteobacteria. Indeed, we find that 30 out of 61 (49%) bacterial DBDs have Bacteria as their taxonomic limit (shared by more than one phylum). These shared DBDs participate in basic carbon source metabolism, e.g. HTH_AraC, LacI and Gnt, as well as in more specific processes, such as FUR (ferric uptake regulator), MerR (mercury resistance) and HTH_8 (virulence gene expression).

Examples of prokaryotic phylum-specific DBDs include WhiB, a DBD specific to Actinobacteria that regulates mycelium formation. The FlhC and FlhD TFs, with Proteobacteria (Gram-negative) as their taxonomic limit, have been shown to be global regulators involved in many cellular processes, including flagella transcriptional activators [11]. On the basis of their restricted phylogenetic distribution and flagella regulation, they might be linked to the Gram-negative's four-support-ring flagella, as opposed to the Gram-positive's two-support-ring flagella. Additional discussions on lineage-specific DBD families and the biological processes they are implicated in are available in the supplementary material online.

## The eukaryotic DBD repertoire is highly specific at the kingdom level

In contrast to the uniform DBD occurrence in Bacteria, Figure 1a shows more distinct expansion patterns among the three main eukaryotic kingdoms: Metazoa (animals), Fungi and Viridiplantae (plants). Indeed, a relatively small proportion (29%) of eukaryotic DBD families have Eukaryota as their taxonomic limit. These eukaryotic families include the zinc finger families, HLH (helix-loop-helix) and bZIPs (basic leucine zippers). In addition, the homeobox family, well known for its role in morphogenesis and animal body development [12], is found throughout eukaryotic organisms, including fungi and plants.

The most notable difference in the Metazoa is between vertebrates and invertebrates. Although the majority of DBDs found in animals are present in both groups, the expansion tends to be less pronounced in the invertebrates. The DBDs with particularly extensive expansion in vertebrates include STAT (signal transduction), T-box (body plan and organogenesis) and p53 (cell cycle arrest and apoptosis). DBDs such as IRF (interferon regulator factor) and Churchill (neural development) are absent from invertebrates, which might reflect the more elaborate immune and nervous systems in vertebrates. In contrast, the Runt and GCM families regulate fundamental developmental processes in both vertebrates and invertebrates, and are equally expanded in both groups.

Metazoa and Fungi are phylogenetically closer and share more DBD families with Viridiplantae (see the supplementary material online). In accordance with earlier work [13], we observed a number of fungal-specific DBDs, including Zn2/Cys6 (Zn cluster), and Copper-fist (copper utilisation). Interestingly, HTH_AraC (arabinose operon regulatory) and FMN (flavin mononucleotide) binding domains are exceptional cases of bacterial DBDs broadly found across Fungi. These families have been shown experimentally to be involved in sugar uptake [14] and sporulation regulation [15] in Bacteria. Their functionality in Fungi has yet to be investigated. Plants possess a number of mainly plant-specific DBDs, such as AP2 (activation of defence genes) and SBP (flowering development).

Apart from the three major kingdoms, we observe an interesting DBD occurrence in the unicellular eukaryote *Monosiga brevicollis,* a marine choanoflagellate that is thought to be the closest sequenced unicellular relative of animals [16]. Earlier studies showed that the species contains a considerable amount of signalling components in common with animals [17]. Besides the more elaborate signalling machineries, uni- to multicellular transitions also require a greater amount of components that contribute to the more complex genetic regulatory networks in functionally diverse cell types [18]. One possible way to enhance the regulation capacity is by recruiting novel sets of TFs. We observed DBDs common to the fungi/animal group in *M. brevicollis*, and many DBDs specific to animals (MB, Figure 1a). Among these DBDs there are families that regulate animal-specific processes such as STAT (signal transduction), p53 (apoptosis) and Tub (nervous system development), as well as those involved in more general pathways like E2F/DP (cell cycle).

In addition, we observed several interesting DBD occurrences in rare protist genomes (Figure 1a). For example, STAT and WRKY were detected in *Dictyostelid* [19,20] and are detected in our dataset. We note the occurrence of two DBDs thought to be plant-specific DBDs in protists. Apart from AP2, which was detected in apicomplexa [21], we discovered a rare presence of the zinc finger LSD1 in many euglenozoa for the first time. Our understanding of transcriptional regulation and the number of sequenced genomes in these protists are, however, still very limited.

## Variety in domain architecture adds complexity to TF structures

**Figure 2:**
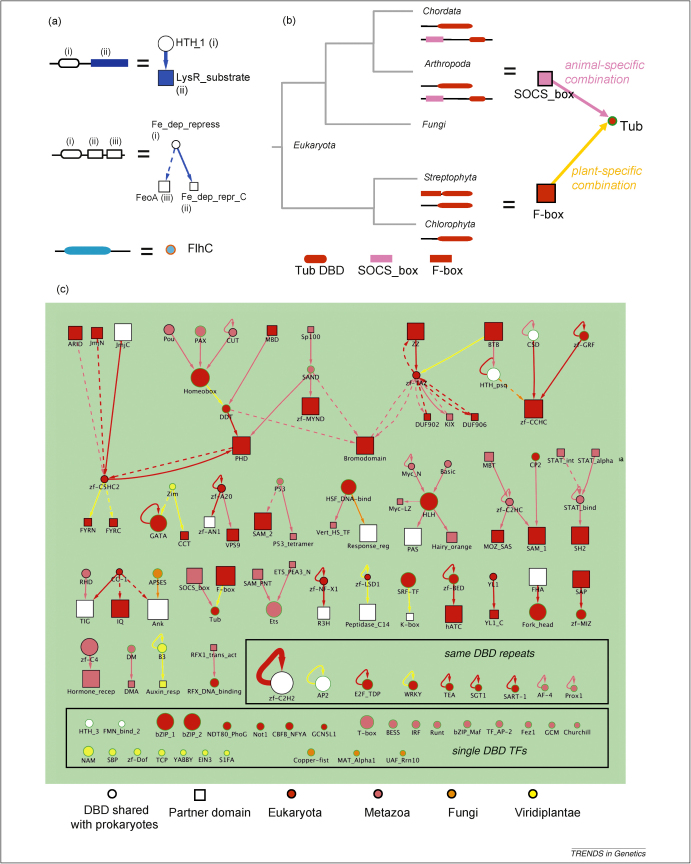
Network representation of DBD families and partner domains. **(a)** Examples of network representation of bacterial TF architectures. DBDs are shown as oblongs in protein chains and as circular nodes in our network representation. Partner domains are shown as rectangles in protein chains and as squares in the network representation. DBDs, e.g*.* HTH_1 and Fe_dep_repress, and their adjacent partner domains, e.g. LysR_substrate and Fe_dep_repr_C, are linked by unbroken arrows, pointing in the N- to C-terminal orientation. Broken arrows connect DBDs and partner domains that occur in the same TF chain but are not adjacent to DBDs, e.g. FeoA. Numbers on the top of each domain indicate its order from N- to C-terminus. Node sizes and arrow thickness are proportional to the abundance of domains and their combination, respectively. Coloured nodes and arrows indicate phylum-specific domain occurrence and domain combination, obtained from the taxonomic limit method described in Box 1 (e.g*.* the blue arrow linking HTH_1 to LysR_substrate indicates the combination is common to all Bacteria). Colour codes are as described for Figure 1. A white node means that the DBD is shared with other superkingdoms, e.g. HTH_1 and Fe_dep_repress are shared by Archaea. DBDs that occur alone as single-domain TFs in more than 25% of all their architectural patterns have orange borders, e.g. FlhC (see the supplementary material online for a complete bacterial TF architectural network and statistics used to generate the network). **(b)** Lineage-specific TF architectures in eukaryotes. A eukaryote-specific Tub DBD (represented by red oblongs in protein chains and by a circular node in our network) has distinct domain combinations in animals and plants. Although the Tub DBD occurs in single-domain TFs without a partner throughout eukaryotic species (green border), in animals, it occurs also C-terminal to the animal-specific SOCS_box (shown as a pink square node, a pink arrow indicates an animal-specific architecture). In contrast, the Tub DBD co-occurs with the F-box domain in plants, a eukaryote-specific partner domain (shown as a red square). This combination is observed exclusively in land plants (linked by a yellow arrow). (**c**) A network representing eukaryotic TF architectures. All architectures that occur in more than 5% of TFs in each DBD family are shown. DBDs that occur alone as single-domain TFs in more than 25% of all their architectural patterns have green borders. We observed the repetition of the same DBD within a TF (self-looping arrow) in 29% of eukaryotic DBDs, whereas DBD repeats in prokaryotes were observed in only one bacterial DBD, HTH_AraC.

TFs have DBDs as core components and often contain other protein domains of different functions, which we term partner domains. In Figure 2, we use a network-style representation to provide an overview of the most commonly occurring TF architectures (those occuring in >5% of TFs in each family). Using our taxonomic limit method to infer the origins of the DBD–partner domain combinations, we observed many lineage-specific TF architectures on top of lineage-specific DBDs (different coloured arrows connecting domain nodes in Figure 2).

The combinations between DBDs (circular nodes) and their partner domains (square nodes) in bacterial TFs are mostly (31 out of 44) shared by more than one bacterial phylum (see supplementary material online for a complete bacterial TF architectural network). For instance, HTH_1 (lysR family), the most abundant DBD in prokaryotes, is always located upstream of the LysR substrate-binding domain (Figure 2a). The blue arrow linking the two domains indicates that this architecture is broadly conserved in all Bacteria. A few TF architectures, such as in Fe_dep_repress, are conserved in all Bacteria as well as in Archaea. In agreement with earlier observations [22], we note that bacterial partner domains function predominantly in small molecule binding or two-component signal transduction. Interestingly, we observed that 16 out of 19 phylum-specific DBDs occur in single-domain TFs without a partner, e.g. FlhC. This is possibly because they emerged relatively recently and have not had sufficient time to combine with other domains to form more elaborate architectures.

Specific DBD–partner domain combinations are observed in animals, fungi and plants. The eukaryotic-specific Tub family, for instance, occurs in a single-domain TF in more than 25% of eukaryotic TFs (green border node). It occurs also downstream of the SOCS_box domain only in animals, and co-occurs exclusively with F-box in plants (Figure 2b). This family is absent from Fungi. These findings suggest that some eukaryotic DBDs have gained new regulatory modes by combining with different partner domains in different kingdoms.

Another distinctive feature of eukaryotic TF architectures not found in prokaryotes is the repetition of the same DBD family within a single TF chain (self-looping arrows in Figure 2c). DBD repeats are found in 22 out of 77 (29%) eukaryotic DBDs, mostly in the zinc fingers. Other DBDs in this category include CUT, E2F/DP and Tea. Additionally, AP2, B3 and WRKY are families that exhibit repeats exclusively in plants (yellow self-looping arrows). The function of repeated DBDs in eukaryotic TFs is most likely to boost the specificity and diversity of motif recognition at TF–DNA interfaces by increasing the number of possible DNA-binding sequences from a limited number of DBD families [23].

The partner domains in eukaryotic TFs have more diverse functions than those in Bacteria, and the commonest function is to mediate protein–protein interaction and dimerisation. This is thought to be important to the formation of composite protein modules, a crucial step towards combinatorial regulation. Examples of these families include BTB, Bromodomain, SAM, ANK and hATC.

## Concluding remarks

DBDs are essential to all sequence-specific TFs because they regulate the specificity of TF–DNA binding, which in turn governs differential expression and determines physiological diversity in different species across the tree of life. With this analysis of conserved and lineage-specific DBDs, and TF architectures using our new method for inferring taxonomic limits, we contribute new insights into the global picture of the TF repertoire and its evolution. Our findings can facilitate the experimental design of high-throughput studies on transcriptional regulators, e.g. Refs [24–28]. In addition to providing an improved understanding on how different DBD families are related, our taxonomic inference methods can be applied to other protein domains apart from DBD families.

We demonstrate a limited conservation of DBD families between prokaryotes and eukaryotes. Only 15% of known DBDs have cellular organisms as their taxonomic limit, as opposed to 33% of all Pfam domains. Lineage-specific DBD repertoires can be seen at the eukaryotic kingdom level: only 29% of eukaryotic families are shared by more than two superkingdoms. Prokaryotic DBDs are less specific to the major bacterial phyla, with 49% of families being shared. In addition to DBD, the variety in DBD and partner domain combination adds another level of complexity to TF structures. The specific DBD families and TF architectures in different lineages can be used as signatures for the genetic regulatory circuits in diverse phylogenetic groups. Knowledge of the phylogenetic distribution of DBD families and their domain combinations can improve methods for remote homology detection [29,30] and advance the discovery of new TFs in genomes.

## Figures and Tables

**Figure I:**
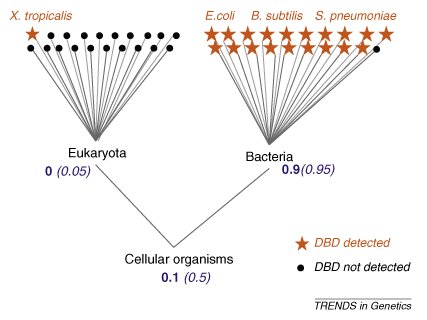
Examples of the taxonomic limit and conservation density calculations for a DBD family using a simplified tree of life. Suppose a DBD family is detected in one TF per genome in one out of 20 eukaryotic genomes and 19 out of 20 bacterial genomes. As there are 20 genomes containing the DBD of interest, there are 190 possible ways of picking a pair of these 20 genomes (binomial coefficient of ^20^*C*_2_, i.e*.* choose 2 from 20). Out of these 190 pairs of genomes, 171 have Bacteria as their LCA. The frequency fraction of Bacteria is 0.9 (171/190), which is higher than that at the cellular organisms node of 0.1 (19/190). The frequency fraction ratio of cellular organisms over Bacteria is 0.11 (0.1/0.9), less than the cut-off threshold of 0.2. The method consequently identifies Bacteria as the taxonomic limit and regards the DBD found in the eukaryotic genome of *Xenopus tropicalis* as contamination. The taxonomic conservation density at the bacterial node is 0.95 (19/20), suggesting that the DBD emerged from the same speciation event rather than from horizontal gene transfer. The frequency fraction and conservation density at each node are shown in bold and bracketed italics, respectively.

## Box 1 Taxonomic limits of DBD families

We have developed an automatic method for inferring the origins of DBD families by combining DBD occurrence in different species with taxonomic information. Although there are similar methods (e.g. Refs [31–33]) that use protein content profiles and species trees to reconstruct evolutionary scenarios, they are not identical with our method and are not used for the same purpose (see the supplementary material online for a detailed discussion).

To obtain a taxonomic limit for a particular DBD family *D*, we first collected all species that have the DBD family detected in their genomes and computed *T*_*D,i,*_, the number of TFs containing the family *D* (*t*_*D,i*_)*,* normalised by the number of genes (*G*_*i*_) in species *i* (Equation I). On the basis of the NCBI taxonomic tree, the last common ancestor (LCA) between each species and all other species that share the DBD of interest was derived. This step was repeated for all possible pairs of species, in an all-against-all fashion (all possible pairs of *i,j* species that contain family *D*). For each pair of species *i,j*, the average number of TFs containing family *D (T*_*D,i,j*_) was computed (Equation II).

(I) $$T_{D,i}=\frac{t_{D,i}}{G_{i}}$$

(II) $$T_{D,i,j}=\frac{T_{D,i}+T_{D,j}}{2}$$

(III) $$F_{D,X}=\frac{\sum_{i,j}T_{D,X,i,j}}{\sum_{i,j}T_{D,i,j}}$$

We defined the frequency fraction of a taxonomic node *X* (*F*_*D,X*_), as the ratio of the sum of normalised TFs containing family *D,* sharing the LCA at node *X* ($\sum_{i,j}T_{D,X,i,j}$), over the sum of all normalized TFs containing family *D* in all taxonomic nodes ($\sum_{i,j}T_{D,i,j}$) (Equation III)*.* We identified the most frequent LCA (highest frequency fraction node) to be the taxonomic limit of this DBD family. However, the bias due to different numbers of genomes in different branches (e.g. Proteobacteria dominate Bacteria) might decrease the accuracy of taxonomic limit estimation. We corrected the estimation by shifting the taxonomic limit to the parental node if its frequency fraction over the highest frequency fraction was greater than a cut-off of 0.2 (see the supplementary material online for the calibration of the method and cut-off threshold).

In addition to the taxonomic limit, we calculated the fraction of species containing the DBD over the total number of species under the taxonomic limit. We termed this the taxonomic conservation density. DBD families that emerged from the same speciation event should be detectable in most of the children species (taxonomic conservation density close to 1). In contrast, the DBDs that are observed sporadically in taxonomically distant lineages (small conservation density), are likely to have been disseminated through horizontal gene transfer or have gone through multiple gene loss events. Figure I demonstrates how the method operates using a simplified taxonomy tree.
