# Supplementary material for: Lineage-specific expansion of DNA-binding transcription factor families
Source: Trends Genet. 2010 Sep;26(9-3):388–93. doi: 10.1016/j.tig.2010.06.004 (PMC2937223; doi:10.1016/j.tig.2010.06.004)
Supplement: Supplementary file 1 [file mmc1.pdf]

Online Supplementary Material:

Lineage-specific Expansion of DNA-binding Transcription Factor Families

Varodom Charoensawan, Derek Wilson, and Sarah Teichmann

MRC Laboratory of Molecular Biology

Cambridge, CB2 0QH, UK

Emails: Charoensawan, V. (varodom@mrc-lmb.cam.ac.uk) and Teichmann, S.A. (sat@mrc-lmb.cam.ac.uk)

Project website

http://www.mrc-lmb.cam.ac.uk/genomes/varodom/DBD_analysis_supplementary/

Table of Contents

1. Genomes used in this analysis 2
2. DBD families used in this analysis 3
3. DBD expansion heatmap 3
4. Taxonomic limits and conservation densities 5
   a. Estimating taxonomic limits of DBD families 5
   1. Calibration of the taxonomic limit method and cut-off threshold 7
   c. Taxonomic conservation densities and Monophyletic clades 10
   d. Examples of taxonomic limit and conservation density calculations 12
   e. Taxonomic limit method and previous literature 14
5. Network representation of TF domain architectures 17
6. Additional discussion 17
   a. Conserved and lineage-specific DBDs in prokaryotes 19
   b. Conserved and lineage-specific DBDs in eukaryotes 21
   c. From uni- to multicellular eukaryotes: additional DBD families emerge 22

1. Genomes used in this analysis

Genomes used in this analysis were taken from *the DBD database* [1], which contains TF annotation of more than one thousand completely sequenced genomes from diverse lineages across the tree of life. A representative non-redundant group of organisms was selected from the DBD database to represent the DBD occurrence expansion in different lineages in the heatmap.

One possible source of bias of using all the genomes available is the variance in numbers of genomes in different lineages. For example, certain types of pathogenic bacteria and fungi are important from a medical and agricultural point of view and have been intensively studied. To minimise this bias, only the most well-characterised strain was selected to represent each particular species. Other redundant strains were excluded from this list. For instance, *Escherichia coli* K12 and *Staphylococcus aureus* NCTC 8325 were used to represent *Escherichia coli* and *Staphylococcus aureus*, respectively. For eukaryotic genomes, only the longest transcripts per gene were considered. We note that an analysis of splice-variants across many genomes is confounded by the heterogeneity of the data available for different organisms. For instance, mouse is extremely well-characterised, while chimpanzee is not. As a result, alternative splicing was excluded entirely from this study. This also allows the numbers of eukaryotic TFs to be compared with the bacterial TFs, which do not contain splice-variants.

To ensure a clear and meaningful DBD expansion in our heatmap, we further refined our genome list by filtering out species with a small number of predicted TFs which exhibit negligible expansion. Organisms that possess less than 50 predicted TFs were excluded from this analysis. The majority of these species are obligate parasites such as *Plasmodium* (eukaryotic microbes), *Mycoplasma* and *Chlamydophila* bacteria. Other poorly characterised eukaryotic genomes were also removed manually if bacterial contamination was detected. These contaminated genomes displayed a great large number of bacterial-specific DBD families that are not observed in other closely related species. The eukaryotic genomes removed during this process include *Apis mellifera* (honey bee), *Ricinus communis* (castor bean), *Capitella sp.I* (segmented worm), *Popolus trichocapa* (western balsam poplar), *Physcomotrella patens subsp. patens* (moss) and *Xenopus tropicalis* (frog). The contamination in the frog genome in particular, has been observed before [2] and the honey bee genome has been removed from Ensembl. After this species refinement, the final number

of organisms was 538, comprising 160 from Eukaryota, 30 from Archaea, and 348 from Bacteria. A table containing a complete list of genomes can be obtained from the authors' project website.

2. DBD families used in this analysis

The DBD families used were also obtained from the DBD database. The prediction was performed based on the presence of DBDs, from two HMM libraries: SUPERFAMILY and Pfam. SUPERFAMILY HMM models are designed to identify members of superfamilies, based on the domain definition of Structural Classification Of Proteins, SCOP [3]. Since protein domain members in SCOP superfamilies tend to be functionally diverse, manual curation in the DBD database was done at the SCOP family level instead [4]. Moreover, it has been shown that many SCOP families have homologous connections to Pfam families [5]. For these reasons, the analysis was performed for all Pfam and SCOP family DBDs. SCOP family (derived from SUPERFAMILY version 1.69) and Pfam (version 18) DBD lists are manually curated and undergo occasional refinement. The latest curated DBD lists were used in this work. Although we show the results from analysing 131 Pfam domains classified as DBDs in this article, what we discuss here for Pfam DBDs also apply to 87 SCOP families manually classified as DBDs in the DBD database. Some of the results for the SCOP family DBDs are documented in this Supplementary Material.

3. DBD expansion heatmap

To survey the presence and absence of DBDs in different lineages, we collected the number of TFs containing each DBD family in each of 538 representative organisms obtained from the refinement procedure explained above. We divided this absolute TF number by the number of genes in each species and present the result in a single-colour heatmap (see Figure 1, high-resolution version can be obtained from our project website). It is clear from this heatmap that the number of DBD families shared between prokaryotes and eukaryotes is very small, but the contractions of DBDs are not visualised. The table containing number of TFs in each DBD family normalised by total number of genes in each genome, which was used to generate this heatmap, can be obtained from our project website.

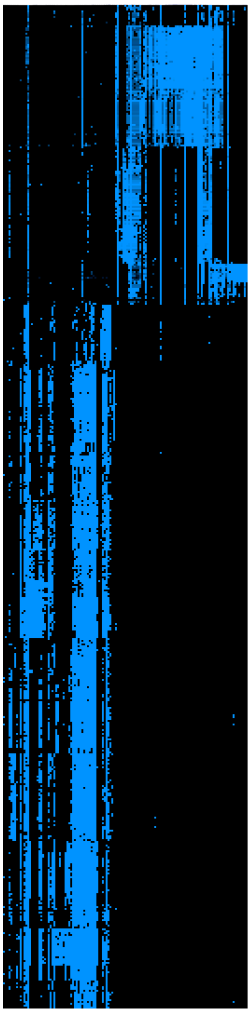

Figure 1 Single-colour heatmap of absolute TF counts (containing Pfam DBDs) normalised by number of genes. The heatmap reviews limited number of DBDs shared by prokaryotes and eukaryotes but the contractions of DBDs are not visualised.

To further improve the presentation, we developed a two-colour heatmap that represents the expansion, as well as contraction/depletion within a particular DBD family. To do so, we computed Z-scores of TFs containing DBDs across all species. For a particular DBD family *D* in species *i*, we counted the total number of TFs containing the DBD family, $t_{D,i}$. As before, the transcription factor counts were normalised by the number of genes in that species, G_i (Equation 1). This is to minimise the bias due to the differences in number of genes in diverse species. We refer to this as normalised TF counts, $T_{D,i}$. For the DBD family *D*, we calculate the Z-score, $Z_{D,i}$, of the normalised count of TFs, containing this DBD in species *i*. This Z-score represents the relative expansion or contraction in different lineages, compared to all other species as described in Equation 2

$$T_{D,i} = \frac{t_{D,i}}{G_i} \quad (1)$$

$$Z_{D,i} = \frac{T_{D,i} - \overline{T_D}}{SD_D} \quad (2)$$

where $\overline{T_D}$ is the mean of TFs containing DBD family D across all species and SD_D is the standard deviation.

Z-scores of all DBD families in all species were visualised as a heatmap using the Genesis 1.7.2 software package [6]. Heatmap columns correspond to DBD families, hierarchically clustered using the complete linkage method and Pearson correlation. Rows correspond to species, ordered according to the NCBI taxonomic tree. The NCBI taxonomy is a expertly curated organism hierarchy which contains more than 300,000 species [7]. Since the taxonomic tree contains more species than any current phylogenetic tree and is manually curated, we preferred it over available phylogenetic trees. A positive Z-score indicates that the DBD is relatively highly expanded in that species and is shown in orange. A negative Z-score represents DBD contraction in the genomes and is shown in blue. High resolution heatmaps for both Pfam and SCOP family DBDs, with family and species name labelled, can be obtained from our project website. Although the distribution of each DBD may not be strictly Gaussian, it is clear that a high relative abundance of DBDs gives positive Z-scores (orange), while depletion corresponds to negative Z-score (blue). This representation has been successfully used before to explain expansion and contraction of DBD families by us and others [1, 8].

4. Taxonomic limits and conservation densities

a. Estimating taxonomic limits of DBD families

To obtain a "Taxonomic limit" for a particular DBD family D, we first collected all species which have the DBD family predicted in their genomes. Based on the NCBI taxonomic tree, the "Last Common Ancestor (LCA)" between each species and all other species that share the DBD of interest was derived. This step was repeated for all possible pairs of species, in an all-against-all fashion (all possible pairs of i,j species which contain domain D). For each pair of species i,j, the average number of normalised TFs containing domain D ($T_{D,i,j,}$) was computed (Equation 3). We termed the "Frequency fraction" of a taxonomic node X ($F_{D,X}$), the ratio of the sum of normalised TFs containing domain D, sharing the last common ancestor X, over the sum of all normalised TFs containing domain D (Equation 4). In many

cases, the described procedure successfully identified the most frequent LCA (highest frequency fraction nodes) as taxonomic limits of DBD families and regarded DBDs detected in a small number of genomes in other branches as contaminations or false hits. Note that this method was used to identify the taxonomic limits of DBD families, by applying to all genomes in the DBD database, not just the 538 genomes in the heatmap. The taxonomic limits of partner domains and domain architectures in TFs were obtained using the same methodology.

$$T_{D,i,j} = \frac{T_{D,i} + T_{D,j}}{2} \qquad (3)$$

$$F_{D,X} = \frac{\sum_{i,j} T_{D,X,i,j}}{\sum_{i,j} T_{D,i,j}} \qquad (4)$$

($\sum_{i,j} T_{D,X,i,j}$ is the sum of normalised TFs of all pairs of species containing domain *D,* sharing the lowest common ancestor *X* and $\sum_{i,j} T_{D,i,j}$ is the sum of all normalised TFs of all pairs of species containing domain *D* in all lowest common ancestors)

One drawback of this approach is its dependency on the numbers of publicly available genomes in different lineages. The method might not accurately pinpoint the true taxonomic limit when the number of species in closely related branches varied greatly. Instead of identifying the true taxonomic limit, the method might pick up one of its child nodes that dominates the genome list (such as Fungi/metazoa which dominate eukaryotic genomes and Proteobacteria which dominate bacterial genomes). By taking the highest frequency fraction nodes as the taxonomic limits for all the DBD families and discarding the DBD occurrences in other branches, the method might underestimate the amount of horizontal gene transfer, which is especially important in shaping the phylogenetic profiles of prokaryotes [9, 10]. Consequently, the trade-off between contaminations and horizontal gene transfer is a crucial issue to the inference taxonomic limit inference.

1. Calibration of the taxonomic limit method and cut-off threshold

To correct the taxonomic limit assignments of these DBD families with potential horizontal gene transfer, we found that the taxonomic limit needed to be shifted down to the parental node. The taxonomic rank of the parental node to be shifted to should be close to the incorrectly assigned child node and its frequency fraction should be large enough. After a careful manual investigation, we discovered the taxonomic limit correction returned the most accurate results when the parental node to be shifted to was not more than 5 taxonomic ranks above the node with the highest frequency fraction. At the same time, the ratio of the frequency fraction of the node to be shifted to, to the highest fraction (hereby called "Frequency fraction ratio"), was greater than the cut-off of 0.2. We manually inspected and found that the DBD families that have this ratio less than 0.2 were indeed bacterial contaminations. The bacterial-specific DBDs that showed contamination traces in Eukaryota include HTH_1, FUR, GntR and MerR.

In addition to the manual inspection of the bacterial contaminations in eukaryotic genomes, we assessed this cut-off threshold more systematically by investigating the number of taxonomic limits assigned to different taxonomic groups, using different cut-offs (Figure 2). The higher the cut-off, the more stringent and narrower the taxonomic limit assignment. For example, a cut-off of 0 means any DBD detected in any genome is regarded as a true hit, even when it is found in only one genome in the superkingdom. Tables describing the taxonomic limits of all 131 DBD families obtained using different cut-offs from 0-0.4 are provided on our project website. Here we manually investigate the DBD families where their taxonomic limits change when the cut-off is lowered from 0.2 (currently used by us) to 0 (most liberal cut-off).

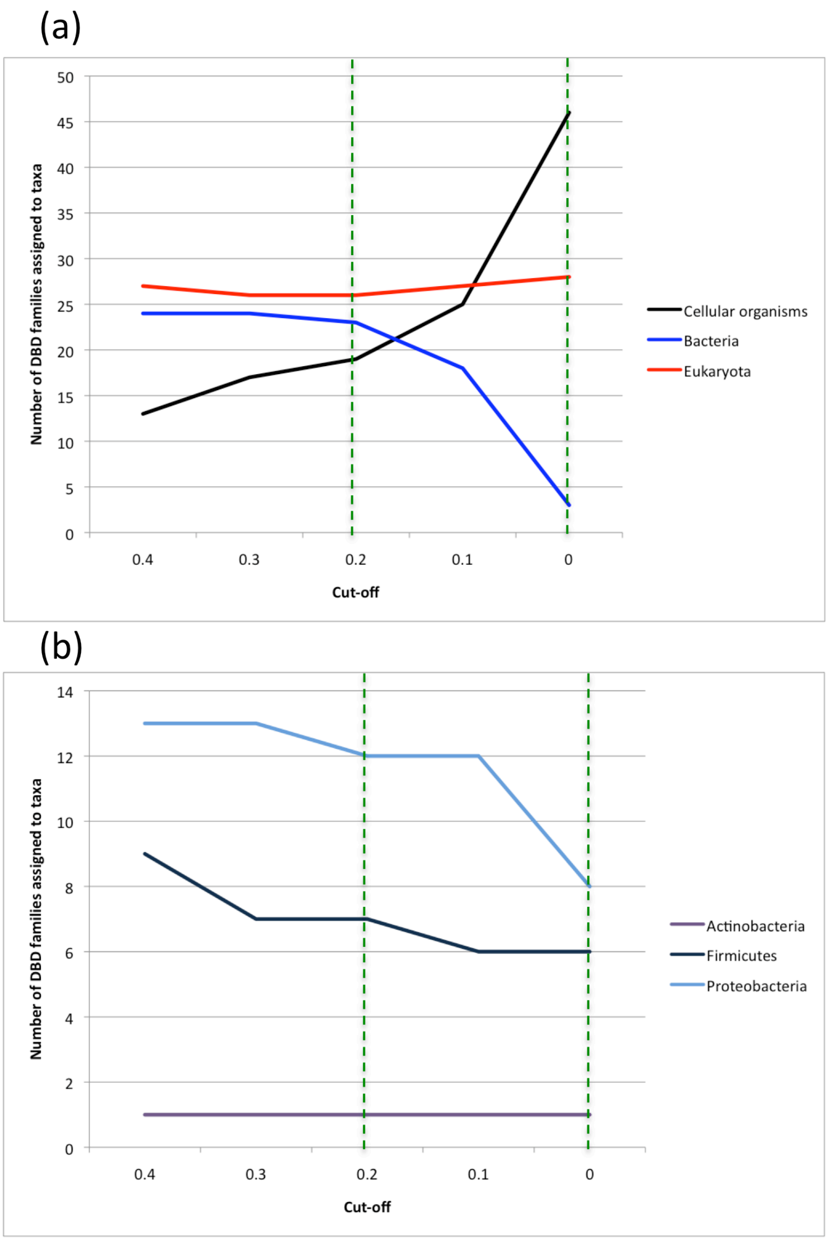

Figure 2 The numbers of taxonomic limits assigned to different taxonomic nodes using different cut-offs from 0.4 (most stringent) to 0 (most liberal). **(a)** The number of taxonomic limits assigned to Eukaryota is relatively unchanged by varying the cut-off used, whereas the number of taxonomic limits assigned to Bacteria drops significantly when the cut-off is 0 because there are many bacterial-specific DBDs found in a small number of contaminated eukaryotic genomes. As a result, the number of DBDs assigned to "cellular organisms" increase when the least stringent cut-off is used **(b)** Within the bacterial genomes, only four DBD families (FlhC, FlhD, ROS_MUCR, and GcrA) have their taxonomic limits switched from Proteobacteria to "cellular organisms" when the cut-off is lowered to 0. This is also likely due to the bacterial contaminations in eukaryotic genomes. We included a complete list of the taxonomic limits of all DBD families when different cut-offs were used as a supplementary file available from our project website.

(i) Between Eukaryota and Bacteria

As shown in Figure 2a, as we lowered the cut-off from 0.2 to 0, we observed many DBDs that are well-known for their regulatory roles in bacterial species having their taxonomic limits switched from Bacteria to "cellular organisms". When we investigated their presence in all species, we found that most of these DBDs were detected in the majority of genomes under Bacteria, *i.e.* high conservation density, and only in few TFs in a few genomes under Eukaryota. Examples of these DBDs include MerR, HTH_1, FUR, GntR. These families are repeatedly detected in the same set of eukaryotic genomes including *X. tropicalis,* in which the bacterial contaminations are well-documented (*e.g.* [2]). Most other genomes where the bacterial DBDs present are early assemblies, *e.g. R. communis* (castor bean) and *S. mansoni* (trematode parasite). Our method regards these DBDs occurring in small number in a limited number of Eukaryota as contaminations. We have included a list of these bacterial DBDs and eukaryotic genomes where they are detected as a supplementary file available from our project website.

Despite many instances of bacterial DBDs identified as contamination, our method does regard four DBDs shared by Bacteria and Eukaryota as true hits when the cut-off of 0.2 is used. For example, FMN_bind_2 and HTH_AraC have been experimentally shown to be involved in sugar uptake [11] and sporulation regulation [12] in bacteria, respectively. It is clear, even from the heatmap (Figure 1 in the main text), that they are also detected in more than half of the fungal genomes in our dataset and might have been disseminated through horizontal gene transfer. These two families are also discussed in the main text. The other two DBD families falling in this category are AP2 and KilA-N. All four of these families shared by Bacteria and Eukaryota have "cellular organism" as their taxonomic limits.

(ii) Between Eukaryota and Archaea

The number of archaeal genomes available is very small compared to the bacterial or eukaryotic genomes and thus the presence of DBDs in Archaea is harder to verify. Nonetheless, our method regards a eukaryotic DBD zf-C2H2 found in half of the archaea as true hits and thus assigns "cellular organisms" as the taxonomic limit of the family.

(iii)Between Bacteria and Archaea

DBD family sharing between the two prokaryotic superkingdoms is common and already well-studied [9]. We have already discussed in the main text that a large number of DBD families shared by bacterial and archaeal genomes might be a result of horizontal gene

transfer between the two prokaryotic superkingdoms. As expected, the presence of DBDs in Archaea is harder to confidently verify whether or not they are true hits due to the small number of archaeal genomes as described above. Using a cut-off of 0.2, our method regards most bacterial DBDs found in Archaea as true hits, except for a small number of cases such as LexA_DNA_bind which is found in only one archaeal genome. In this case, our method assigns Bacteria as their taxonomic limit instead of "cellular organisms".

(iv) Within Bacteria

Horizontal gene transfer is known to play a crucial role in shaping the phylogentic profiles of prokaryotes [9, 10]. This corresponds to our taxonomic limit assignments where approximately half of the DBD families found in bacterial species have Bacteria as their taxonomic limits, rather than a more specific bacterial subgroup. However, we also found a number of phylum-specific DBDs. These families are truly phylum-specific because the numbers of DBDs assigned to phyla drop only slightly even when the cut-off was lowered from 0.2 to 0 (Figure 2b). The only four DBDs which have their taxonomic limits switched from Proteobacteria to cellular organisms are FlhC, FlhD, ROS_MUCR, and GcrA. The FlhC, FlhD, ROS_MUCR families are found only in one eukaryotic species *R. communis* and are most likely due to bacterial contaminations during genome sequencing. The GcrA family is only found in one archaeal species, *S. solfataricus* and could also be due to bacterial contamination.

There are no phylum-specific DBDs that have their taxonomic limits switched to Bacteria when the cut-off was lowered to 0. This shows that the number of bacterial phylum-specific DBDs the method estimates is not overestimated, and that horizontal gene transfer within the bacterial species is not underestimated.

(v) Within Eukaryota

Horizontal gene transfer between eukaryotic species is thought to be rare, especially in multicellular organisms [13]. Nevertheless, we have discussed the presence of some animal-specific DBDs in the choanoflagellate *M. Brevicollis,* which is interesting from the point of view of multicellular eukaryotic evolutionary.

c. Taxonomic conservation densities and Monophyletic clades

To estimate the proportion of children species within a taxonomic limit clade that actually contains the DBD family of interest, we calculated the fraction of species containing the

DBD over the total number of species within that taxonomic node. We termed this fraction the "Taxonomic conservation density". "Monophyletic clades" were defined as nodes below the taxonomic limit where at least 98% of their members contain the DBD family of interest (having taxonomic conservation density greater or equal to 0.98). We decided to use this 0.98 cut-off instead of 1 (all children nodes contain the DBD) because we observed in the majority of lineage-specific families, that the assignments of DBDs were always technically missing in a small number of species members within the lineage (Figure 3). This process was used to find all monophyletic clades at taxonomic ranks below the taxonomic limit nodes for all DBD families. A complete table containing the taxonomic limits, conservation densities, and monophyletic clades for all Pfam and SCOP family DBDs can be obtained from our project website.

In addition to the taxonomic limits, this information is also useful for inferring the origin of DBD families and assessing horizontal gene transfer events which might have occurred in the families. DBD families that emerged from the same speciation event should be detectable in most of the children species (taxonomic conservation density close to 1). In contrast, the families that are observed sporadically in taxonomically distant prokaryotic lineages (small conservation density), are likely to have been disseminated through horizontal gene transfer or massive multiple gene loss events have occurred.

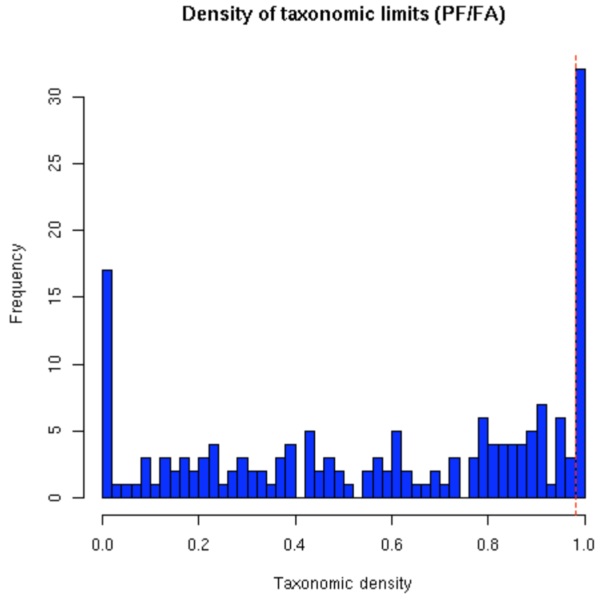

Figure 3 Taxonomic conservation density distribution of Pfam (PF) and SCOP families (FA). The majority of taxonomic limits have conservation densities greater than or equal to 0.98.

d. Examples of taxonomic limit and conservation density calculations

In addition to the discussion on how we assessed the taxonomic limit results when different cut-offs were used, we provide examples of taxonomic limit and conservation density calculations for four DBD families in **Figure 4** (DBD name followed by frequency fraction ratio in the brackets):

i. **MerR (0.14):** The MerR family is known to mediate the mercuric-dependent induction of the mercury resistance operon in bacterial species [14]. The family is detected in most prokaryotic genomes but also in a few eukaryotic genomes, *i.e. X. tropicalis* and *R. communis*). Because the frequency fraction ratio of "cellular organisms" over Bacteria (0.086/0.611 = 0.14) is less than the cut-off of 0.2, our method does not shift the taxonomic limit to "cellular organisms" but instead identifies Bacteria as the taxonomic limit, in line with a previous study suggesting that there is bacterial contamination in some eukaryotic genomes including *X. tropicalis* [2].

ii. **HTH_AraC (0.21):** The HTH_AraC family is related to the arabinose operon regulatory protein AraC in bacteria [11]. The family is present in approximately 80% of bacterial genomes but also in approximately half of fungal genomes. Our taxonomic limit method shifted the taxonomic limit from Bacteria to "cellular organisms" because the frequency fraction ratio of "cellular organisms" over Bacteria (0.112/0.528 = 0.21) is greater than 0.2.

iii. **Homeobox (0.65):** The Homeobox family is well known for its role in morphogenesis and animal body development [15]. Despite the small number of plant genomes available, the Homeobox family is also found in almost all plants. Our method shifted taxonomic limit to Eukaryota because the frequency fraction ratio of Eukaryota over Fungi/metazoa (0.238/0.364 = 0.65) is greater than 0.2.

iv. **zf-C2H2 (0.30):** zf-C2H2 are found in all Eukaryota but are also found in nearly half Archaea. Even though Eukaryota has the highest frequency fraction, the method shifted the taxonomic limit to "cellular organisms" because the frequency fraction ratio of "cellular organisms" over Eukaryota (0.093/0.306 = 0.30) is greater than 0.2.

These examples demonstrate that our taxonomic limit method has the power to distinguish contaminations from true hits. Apart from these four examples, we have included a complete list of the taxonomic limits of all DBD families when different cut-offs between 0-0.4 were used as a supplementary file available from our project website.

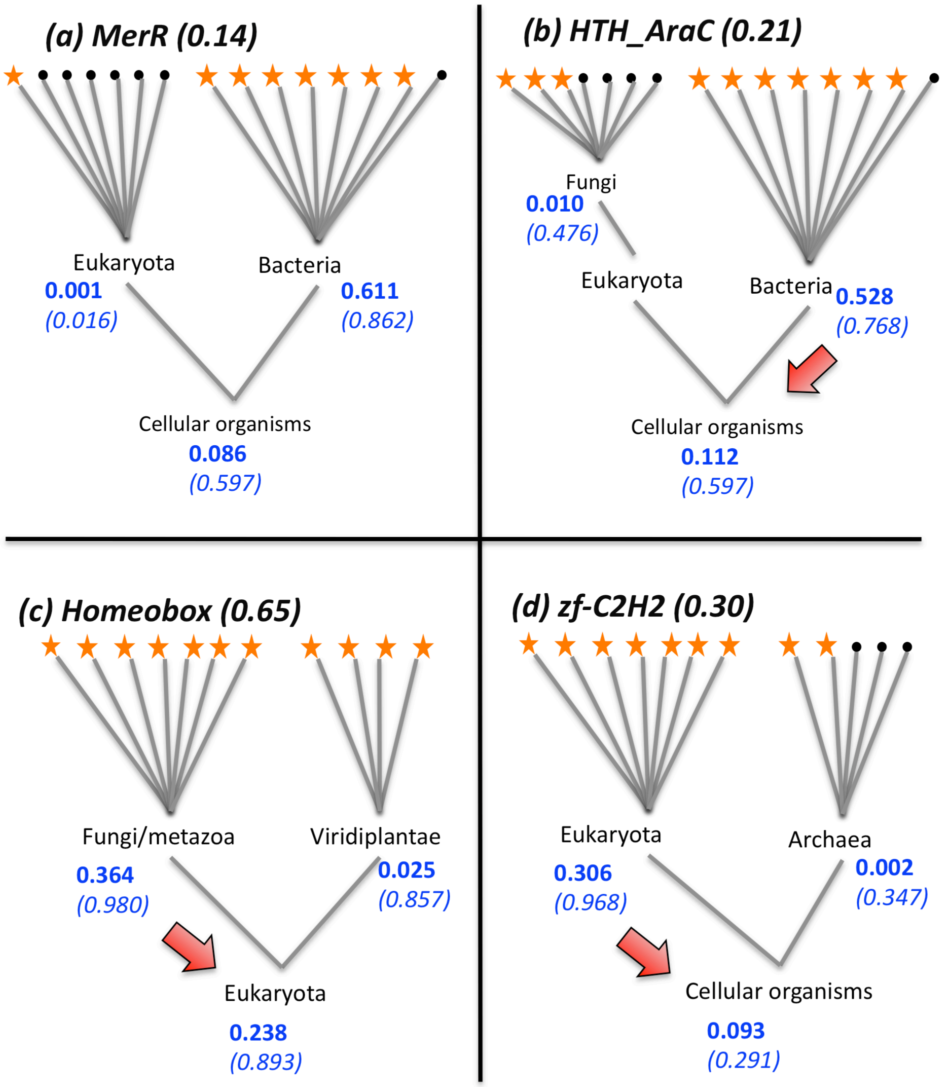

Figure 4 Examples of DBD occurrence on a simplified phylogenetic tree **(a)** The MerR family is detected in most prokaryotic genomes but also in a few eukaryotic genomes. Because the ratio of the frequency fraction at cellular organisms over at Bacteria is less than the cut-off of 0.2, our method does not shift the taxonomic limit to cellular organisms but instead identifies Bacteria as the taxonomic limit **(b-d)** The HTH_AraC, Homeobox, and zf-C2H2 families have the ratio of the frequency fraction of the nodes to be shifted to, to the highest one, greater than 0.2, our method shifts the taxonomic limits to the parental nodes and regards the DBDs found in other branches as true hits. The frequency fraction at each node is shown in bold and the taxonomic conservation density is shown in the brackets and italics.

e. Taxonomic limit method and previous literature

In this section, we provide a detailed discussion of previously published approaches for inferring the evolutionary scenarios of proteins or protein families. Although they might seem similar to our method, they are not identical and are not suitable for our purpose. Here we discuss the strengths of our new taxonomic limit method and compare it to the existing approaches that are most relevant.

Many studies have shown that the phylogenetic relationship of proteins can be inferred by building a phylogenetic tree, either based on sequence similarity or presence/absence profile, (as reviewed by [16]). However, such a tree would only contain the species where the DBD is present, which means gene losses cannot be assessed. Moreover, the trees built using different families are often different. Consequently, we focus on the methods that are, like ours, based on presence/absence profiles of protein families and a reference tree.

To the best of our knowledge, there are only a small number of groups that have combined the gene-content profiles and phylogenetic relationships to reconcile evolutionary scenarios. Koonin and Mushegian [17] and Kyrpides *et al.* [18] were among the first to do so. However, they only focused on the minimal gene set of the last universal common ancestor, which is not relevant to our analysis.

More relevant methods to what we describe were published by Snel *et al.* [19], Kunin *et al.* [20] and Mirkin *et al.* [21]. They all focus on constructing the most parsimonious evolutionary scenarios (gene gain, loss, horizontal gene transfer occurring at all internal nodes) for protein families given a species tree. Although such methods could, in principle, be used to estimate the earliest node where the DBD family became present, none of these methods or papers actually does this. There are also a number of technical and conceptual issues that make them unsuitable for our purpose.

i. All three methods require an accurate bifurcated species tree (branched into two at each internal node). Because their parsimonious algorithms start from terminal (leaf) nodes of the tree and move towards to the root, it is important that the relationships between species are accurate near the terminal nodes. To the best of our knowledge, there is no existing phylogenetic tree that matches the number of genomes in the DBD database (over 1000 genomes to date). We thus decided to use the NCBI taxonomy tree, which provides a manually curated organism hierarchy of more than 300,000

species [7]. However, the taxonomy tree is not bifurcated as more than two species can share the same parental node. For instance, the taxonomic node *Escherichia* is parent to at least 50 *Escherichia* species including *E. coli. K12.* Thus, accurate phylogenetic relationships between species under the same parent might not always be attainable.

Our taxonomic method simply estimated an internal node where any arbitrary pair of species containing the DBD family of interest meets most often (weighted by the number of TFs containing the DBD family). Consequently, the method is robust against uncertain phylogenetic relationships near terminal nodes. Suppose family A is common to all bacterial genomes, even though the relationships near the leaves are unspecific or inaccurate (at genus or family level), our taxonomic method will still identify Bacteria as the family's taxonomic limit because most arbitary species meet most often at the bacterial node. Such an approach works even when the reference tree used is not bifurcated, as in the case of the NCBI taxonomy tree. Additional examples are given in the Supplementary Material.

ii. All three methods were previously implemented in prokaryotes, where horizontal gene transfer HGT plays a crucial role in shaping the phylogentic profiles [9]. One of the major dilemmas involving prokaryotic genome evolution is distinguishing gene gain, gene loss, and horizontal transfer. To address this problem the authors introduced parameters to assess the probability of multiple gene loss events versus HGT events (called GAIN threshold by Kunin *et al.* and HGT penalty by Snel *et al.*). These parameters have been carefully explored for prokaryotic genomes but the same set of parameters will not be suitable for eukaryotic genomes, where HGT contributes to the evolution of genomes at a much lower rate, especially after the emergence of multicellular organisms [13].

iii. The methods published by Snel *et al.* [19], and Mirkin *et al.* [21] are only applicable for a group of proteins where their orthologue definitions are available because additional orthologous information is required. Consequently, they do not suit our purpose where we want to estimate the taxonomic limit of the entire family because its protein members not always being orthologous.

iv. Our method is flexible and not restricted to the taxonomic limits of protein families. It can also be used to estimate when a domain combination between two domains occurred. To the best of our knowledge, this is the first time such an analysis has been done at the domain architectural level.

In summary, our method for estimating taxonomic limits is simple, intuitive, fast and robust to uncertainties of trees near species nodes. We have shown that the method has sufficient power to distinguish contaminations from horizontal gene transfer.

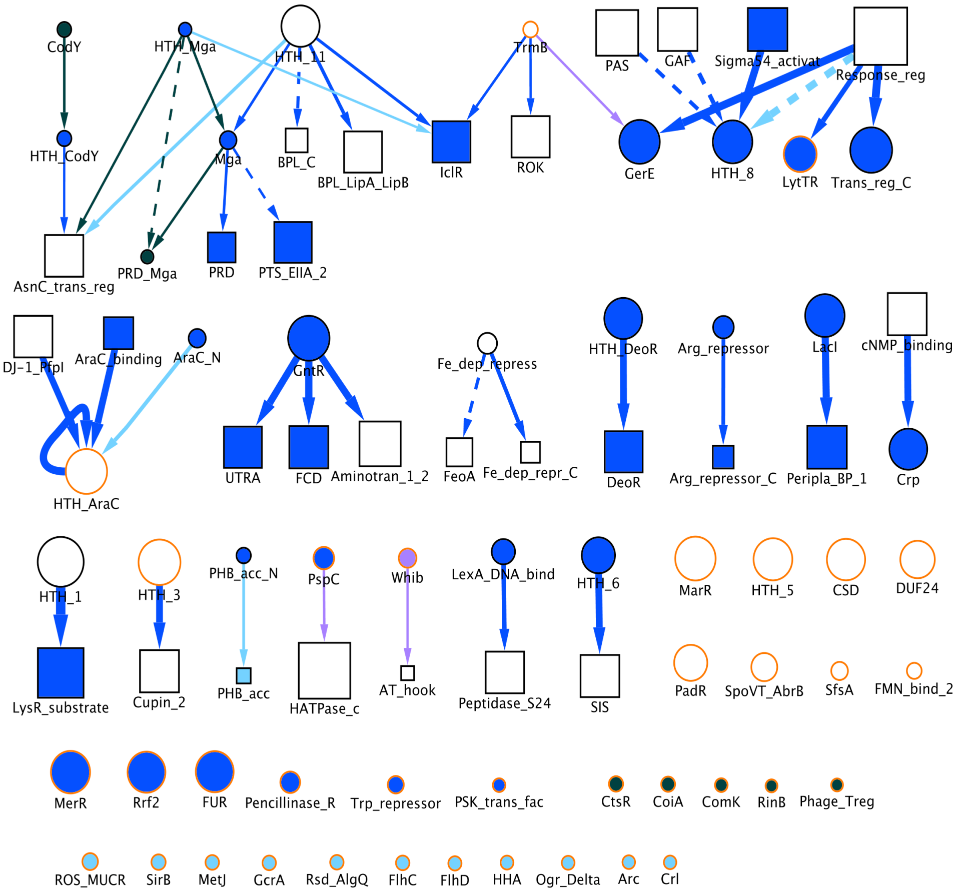

Figure 5 Network representing bacterial TF architectures. Circular nodes correspond to DBDs and squares represent partner domains. Neighbouring domains are represented by solid arrows in N- to C- termini orientation. Dotted arrows represent partner domains which occur in the same TF chain but are not adjacent to DBDs. Node sizes and arrow thickness are proportional to the abundance of domains and their combination, respectively. Coloured nodes and arrows indicate phylum-specific domain occurrence and domain combination, respectively. Colour codes are as described in the main text. White means the DBD is also shared with other

superkingdoms (Eukaryota and/or Archaea). DBDs which occur alone as single-domain TFs in more than 25% of all their architectural patterns have orange borders.

5. Network representation of TF domain architectures

The most common domain architectures of bacterial and eukaryotic TFs are illustrated separately using a network representation. Partner domains and architectures that occur in more than 5% of TFs for each DBD family were gathered and TF architecture networks were generated using Cytoscape 2.6.1 [22]. In a small number of partner domains, their functions (such as topoisomerases and endonucleases) suggest that they are not involved in sequence-specific transcriptional regulation. These domain families were manually removed. Circular nodes correspond to DBDs and squares represent partner domains. Neighbouring domains are represented by solid arrows pointing in N- to C- terminal orientation. Dotted arrows represent partner domains which also occur commonly, but are not adjacent to DBDs. Node sizes and arrow thickness correspond to the abundance of domains and their combination, respectively. Both domain occurrence and domain combinations are labelled according to their taxonomic limits. Nodes and arrows are coloured according to their taxonomic limits, which were derived from the method described above. Colour codes are as described in the main text. DBDs that occur as single-domain TFs for more than 25% of all architectures are highlighted using orange (in bacterial network) or green borders (in eukaryotic network). Tables describing the numbers of domain combinations normalised by numbers of genes, which were used to generate these networks, can be obtained from our project website. In addition to the eukaryotic TF architectural network shown in the main text, a complete bacterial TF network is shown in Figure 5.

6. Additional discussion

In this section we provide additional discussion on conserved and lineage-specific DBD families across the tree of life. The literature on the biological processes the DBDs are implicated in is also extensively documented. In addition to the number of DBDs shared by Archaea, Bacteria, and Eukaryota described by a Venn diagram in the main text, here we provide a simplified taxonomic tree with the number of DBD families and Pfam families having their taxonomic limits assigned to each node (**Figure 6**). These results show that the number of DBDs families having "cellular organisms" as taxonomic limits (15%) is significantly greater than of all Pfam families (33%). This confirms that the repertoires of

DBD families are more lineage-specific than other proteins. In addition, we also show a Venn diagram representing the number of SCOP families classified as DBDs which have taxonomic limits belonging to the three major superkingdoms (Figure 7). Eight out of 88 SCOP families (9%) are shared by the three major superkingdoms, compared to 2% of Pfam DBDs shared.

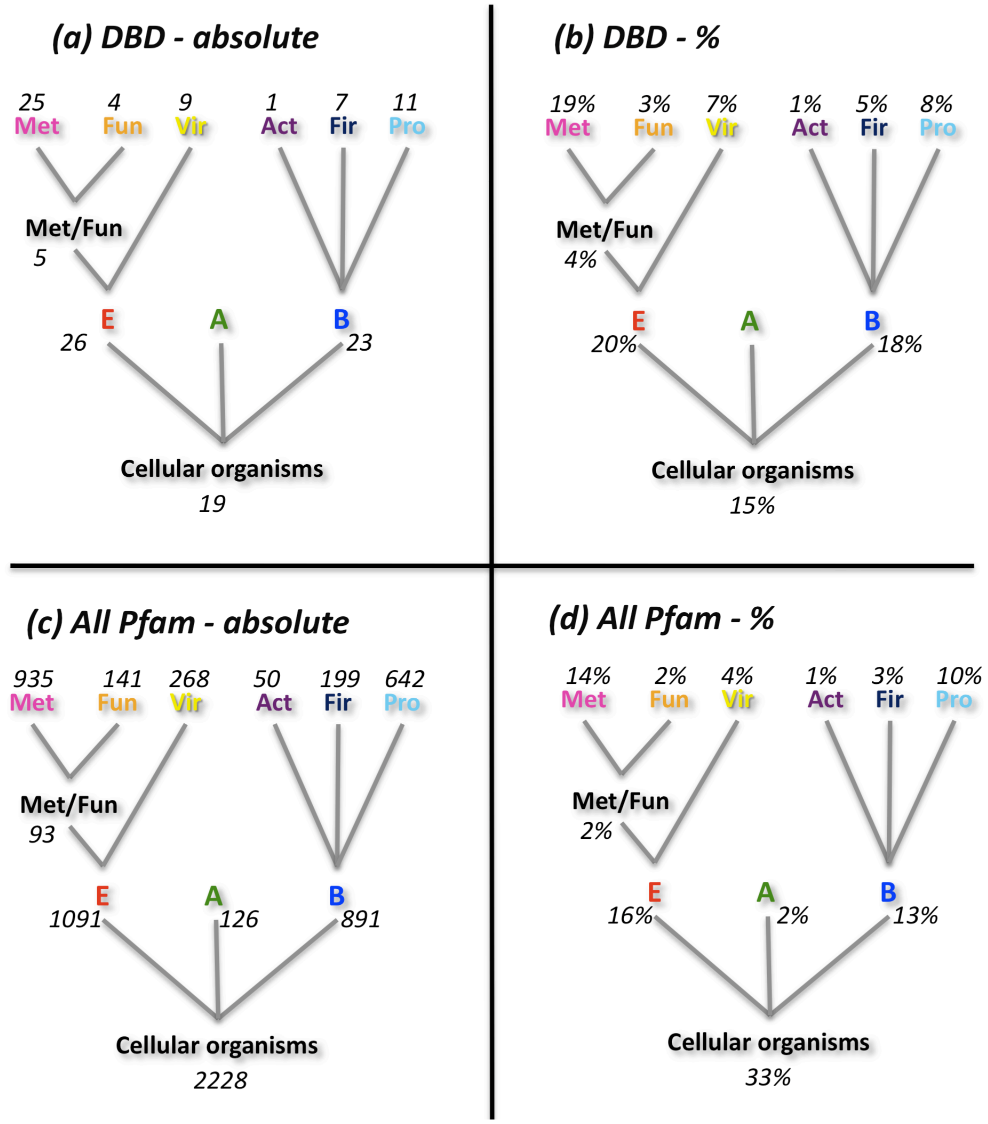

Figure 6 The absolute numbers and the percentages of DBD families and all Pfam families having taxonomic limits assigned to each taxonomic node. The number of DBDs families having "cellular organisms" as taxonomic limits (15%) is significantly greater than for all Pfam families (33%). Eukaryota (E) is divided into three kingdoms: Metazoa (pink), Fungi (orange) and Viridiplantae (yellow). Archaea (A) are in green. Bacteria (B) are labelled using blue shaded colours: Actinobacteria (purple), Firmicutes (navy) and Proteobacteria (pale blue).

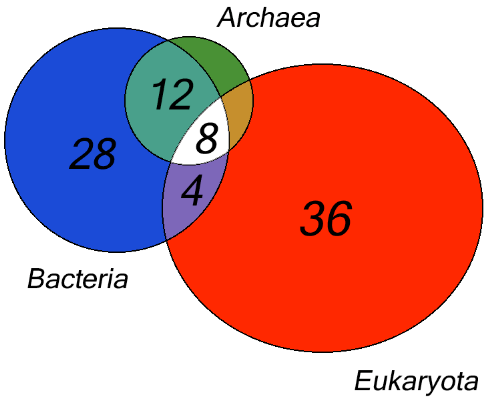

Figure 7 Venn diagram showing conserved and specific SCOP family DBDs in three major superkingdoms. Eight of 88 (9%) of SCOP family DBDs are shared by archaea, bacteria, and eukaryotes, compared to 2% of Pfam DBD families shared by the three superkingdoms.

a. Conserved and lineage-specific DBDs in prokaryotes

Examples of DBDs shared by the two prokaryotic superkingdoms are Iron dependent repressor [23], HTH_5 (arsenic resistance)[24], MarR (antibiotic resistance)[25], NikR (nickel responsive regulator)[26], PadR (phenolic acid metabolism)[27] and TrmB (trehalose/maltose sugar-specific regulator)[28]. These DBD families shared by Archaea and Bacteria regulate specific genes required for adaptation to the environmental stresses except for HTH_1 (LysR family), which participates in amino acid synthesis [29].

The distribution of DBDs in prokaryotes is not only widespread at the superkingdom level but there is also no clearly distinguishable expansion scheme within the three major bacterial phyla: Actinobacteria (purple), Firmicutes (dark blue) and Proteobacteria (light blue). According to Figure 8a, which summarised the taxonomic limits of bacterial DBDs, each phylum seems to possess a small number of phyla specific DBD families but it is apparent that the majority of DBDs are shared by all bacterial lineages.

These conserved DBDs participate not only in basic carbon source metabolism such as HTH_AraC [11], LacI [30] and GntR [31], but also the more specific functions such as FUR (Ferric uptake regulator)[32], MerR (mercury resistance)[14], LexA repressor (DNA repair system)[33], GerE (Lux family, quorum sensing)[34] and HTH_8 (Fis family, virulence gene expression)[35]. These bacterial specific DBDs are all found in more than

60% of bacterial species (conservation densities greater than 0.60) and are most likely inherited from the last common ancestor of all bacterial species.

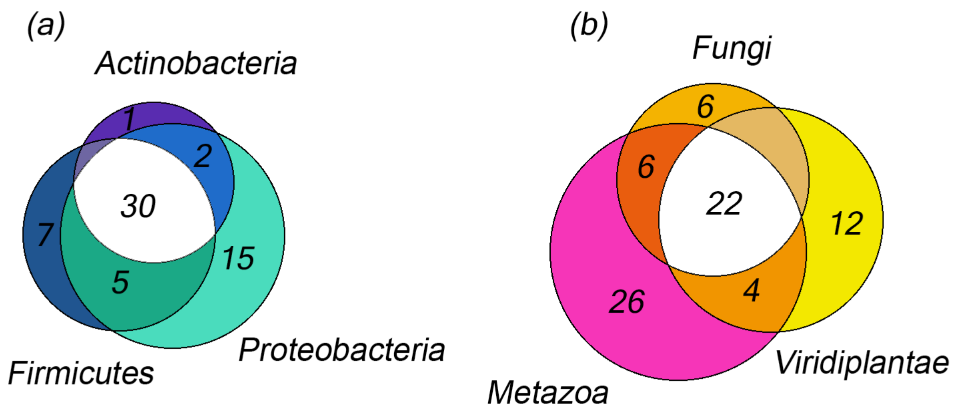

Figure 8 (a) Venn diagram showing the occurrence of Pfam DBD families in three major bacterial phyla and **(b)** in three major eukaryotic kingdoms. Most bacterial DBD families (30 out of 61, 49%) are shared by the three major bacterial phyla, while eukaryotic DBD families are more specific to the major kingdoms (22 out of 77, 29% shared by the three major superkingdoms). Note that there are two DBDs counted in the Venn diagram in the main text but not in these figures. The KilA-N DBD is only observed in *Tricomonas Vaginalis,* a eukaryotic protist which is not part of the animal, fungi or plant kingdom. The Pox_A8 DBD is only observed in *Leptospira borgpetersenii,* a bacteria which is not part of Actinobacteria, Firmicutes or Proteobacteria.

Despite both being Gram-positive bacteria, Actinobacteria and Firmicutes do not share any DBD families beyond the ones that are conserved across all bacteria (Figure 8a). Since most of the Actinobacteria are filamentous, it makes sense that this bacterial phylum has a DBD WhiB [36] specific to mycelium formation regulation. A number of DBDs, which control expression of genes in different pathways, are specific to the Firmicutes: CodY GAF-like domain [37], ComK protein [38], and Firmicute transcriptional repressor of class III stress genes (CtsR)[39], for instance. The sporulation initiation factor (Spo0A) is, however, the most interesting of all. This family reflects the lifestyle of many Firmicutes which reproduce by forming spores in undesirable conditions [40].

Owing to the greater number of completely sequenced genomes available, Proteobacteria possess more phylum-specific DBDs than any other bacterial lineage. The DBD families that fall into this category include Crl (fibronectin binding activators)[41], ROS/MUCR (virulence region repressor)[42] and Met repressor (MetJ, methionine synthesis)[43]. The FlhC and FlhD TFs have been shown to be global regulators involved in

many cellular processes as well as flagella transcriptional activators [44]. They are only present in Gram-negative Proteobacteria but not in Firmicutes and Actinobacteria. The phylogenetic pattern of these DBD families may be linked to the four-support-ring flagella in Gram-negative bacteria, as opposed to the two-support-ring flagella in Gram-positive bacteria.

b. Conserved and lineage-specific DBDs in eukaryotes

In contrast to the disperse DBD occurrence in bacterial species, Figure 1 in the main text demonstrates the distinct expansion patterns between the three main eukaryotic kingdoms: Metazoa (pink), Fungi (orange) and Viridiplantae (yellow), and other unicellular eukaryotic organisms. Metazoans (animals) possess a considerably larger DBD repertoire than the Fungi and Viridiplantae kingdoms (Figure 8b). This reflects the greater morphological complexity and body structures of animals, as well as a potential bias towards the study of animal model organisms.

Only a small proportion of DBD families are ubiquitously present across the eukaryotic superkingdom. These families include the majority of Zinc finger families [45, 46], helix-loop-helix (HLH)[47] and basic leucine zippers (bZIPs)[48]. Surprisingly, the Homeobox family, famous for its role in morphogenesis and animal body development [15, 49], is also found throughout eukaryotic organisms, including fungi and plants.

Distinct expansion schemes are also observed among animal species. The most notable difference is between vertebrates and invertebrates. The majority of DBDs found in metazoans are present in both animal groups, however, the expansion in invertebrates is significantly less pronounced in many DBD families. The DBDs with particularly extensive expansion in vertebrates include: STAT (signal transduction)[50], T-box (body plan and organogenesis)[51] and p53 (cell cycle arrest and apoptosis)[52]. Other DBDs such as Interferon regulator factor (IRF, regulation of immunity)[53], Churchill (neural development)[54] and an oncogene Myc [55], are entirely absent from invertebrates. This is most likely due to more elaborate immune and nervous systems in vertebrates. On the contrary, Runt [56] and GCM [57] families regulate fundamental developmental processes in both vertebrates and invertebrates, and are equally expanded in both groups. It is worth mentioning that the BESS and HTH_psq domains are particularly highly represented in insects, the taxonomic group that dominates the invertebrate genomes.

Being phylogenetically closer to Metazoa, Fungi share more DBD families with animals than with plants (Figure 8b). DBDs which are common to metazoans and fungi but

completely absent in plants include: CP2, Fork head, NDT80/PhoG and Tea domains. DBD occurrence patterns between fungal organisms are more uniform than in metazoans as all fungi possess similar sets of DBD repertoires. In accordance with previous work [58], we observed a number of DBDs detected in Fungi but not in other eukaryotes. Interestingly, not all fungal specific DBDs are restricted to fungal-specific processes. To illustrate the point, the DNA-binding domain of Mlu1-box binding protein MBP1 is mainly involved in regulation of the cell cycle [59]. Zn2/Cys6 (Zn cluster) has many regulatory roles such as in sugar and amino acid metabolism, cell cycle, as well as drug resistance [60]. The Copper-fist domain participates in copper utilisation and stress response processes [61]. MAT alpha1 and APSES, however, do regulate fungal-specific process as they activate mating-type specific genes [62] and regulate yeast-hyphal transitions [63], respectively. HTH_AraC [11] and FMN (Flavin mononucleotide) binding domain [12] are exceptional cases of bacterial DBDs found in many fungi. The families have been experimentally shown to be involved in sporulation regulation and sugar uptake in bacterial species, but their functionality in fungi has yet to be investigated.

Apart from the majority of plant DBD families which are also found in animals and fungi, a set of DBD families are specific to plants including: AP2/GCC-box binding domain (activation of defence genes)[64], SBP (flowering development)[65] and WRKY (pathogen defence and biosynthesis of secondary metabolites)[66]. Additionally, we observe a number of DBD families found in the Streptophyta phylum (land plants) are absent in Chlorophyta (green algae). These families are discussed in the next section.

c. From uni- to multicellular eukaryotes: additional DBD families emerge

Apart from the three major kingdoms, the DBD database also provides TF predictions for many unicellular eukaryotes. Among the unicellular eukaryotic species available, *Monosiga brevicollis* is one of the most interesting organisms as it is the only well-annotated representative of choanoflagellates, the closest known relatives of metazoans [67]. Previous studies on the organism have concentrated on its signal transduction mechanisms and found that the species contained a considerable amount of signalling components in common with animals [68, 69].

Besides the more elaborate signalling machineries, uni- to multicellular transitions also require a greater number of components that contribute to the more complex genetic regulatory networks in functionally diverse cell types [8]. One possible way to enhance regulation capacity is by recruiting novel sets of TFs. By investigating the *Monosiga*

brevicollis genome, we observed not only DBDs common to the Fungi/metazoa group such as Homeobox, HLH, Fork_head and bZIPs, but also many metazoan-specific DBDs not found in fungi. Among the animal-specific DBDs, there are families which regulate animal-specific processes such as STAT (signal transduction), p53 (apoptosis), Tub (nervous system development)[70], as well as those involved in more general pathways like E2F/DP (cell cycle)[71] and Cold-shock domain (CSD, low temperature response)[72].

A shift of lifestyle from single to multicellular organisms also occurred in the Viridiplantae kingdom, in parallel to the Fungi/metazoa lineage. In a similar manner to choanoflagellates, a unicellular green alga *Chlamydomonas* was used to demonstrate the presence/absence of DBD families in unicellular viridiplantae [73]. Our results confirm a number of DBDs shared by Streptophyta (land plants) and Chlorophyta (green algae) previously proposed including: AP2, SBP, WRKY and zf-Dof domains. Apart from NAM (NAC, apical meristem development)[74], we observed additional families which are involved in processes more specific to higher plants and absent from Chlorophyta altogether. Examples of these DBDs are SRF (cell proliferation and differentiation)[75], YABBY (abaxial polarity in lateral organs)[76], EIN3 (ethylene-mediated responses)[77] and TCP (multiple developmental control pathways)[78].

References

1. Wilson, D., *et al.* (2008) DBD--taxonomically broad transcription factor predictions: new content and functionality. *Nucleic Acids Res* 36, D88-92
2. Yang, S., *et al.* (2005) Phylogeny determined by protein domain content. *Proc Natl Acad Sci U S A* 102, 373-378
3. Andreeva, A., *et al.* (2008) Data growth and its impact on the SCOP database: new developments. *Nucleic Acids Res* 36, D419-425
4. Kummerfeld, S.K., and Teichmann, S.A. (2006) DBD: a transcription factor prediction database. *Nucleic Acids Res* 34, D74-81
5. Zhang, Y., *et al.* (2005) Comparative mapping of sequence-based and structure-based protein domains. *BMC Bioinformatics* 6, 77
6. Sturn, A., *et al.* (2002) Genesis: cluster analysis of microarray data. *Bioinformatics* 18, 207-208
7. Benson, D.A., *et al.* (2009) GenBank. *Nucleic Acids Res* 37, D26-31
8. Rokas, A. (2008) The molecular origins of multicellular transitions. *Curr Opin Genet Dev*
9. Aravind, L., and Koonin, E.V. (1999) DNA-binding proteins and evolution of transcription regulation in the archaea. *Nucleic Acids Res* 27, 4658-4670
10. Kunin, V., *et al.* (2005) The net of life: reconstructing the microbial phylogenetic network. *Genome Res* 15, 954-959
11. Saviola, B., *et al.* (1998) Arm-domain interactions in AraC. *J Mol Biol* 278, 539-548
12. Honjo, M., *et al.* (1990) A novel Bacillus subtilis gene involved in negative control of sporulation and degradative-enzyme production. *J Bacteriol* 172, 1783-1790
13. Doolittle, W.F., *et al.* (2003) How big is the iceberg of which organellar genes in nuclear genomes are but the tip? *Philos Trans R Soc Lond B Biol Sci* 358, 39-57; discussion 57-38
14. Helmann, J.D., *et al.* (1989) Homologous metalloregulatory proteins from both gram-positive and gram-negative bacteria control transcription of mercury resistance operons. *J Bacteriol* 171, 222-229
15. Pavlopoulos, A., and Akam, M. (2007) Hox go omics: insights from Drosophila into Hox gene targets. *Genome Biol* 8, 208
16. Wolf, Y.I., *et al.* (2002) Genome trees and the tree of life. *Trends Genet* 18, 472-479
17. Mushegian, A.R., and Koonin, E.V. (1996) A minimal gene set for cellular life derived by comparison of complete bacterial genomes. *Proc Natl Acad Sci U S A* 93, 10268-10273
18. Kyrpides, N., *et al.* (1999) Universal protein families and the functional content of the last universal common ancestor. *J Mol Evol* 49, 413-423
19. Snel, B., *et al.* (2002) Genomes in flux: the evolution of archaeal and proteobacterial gene content. *Genome Res* 12, 17-25
20. Kunin, V., and Ouzounis, C.A. (2003) GeneTRACE-reconstruction of gene content of ancestral species. *Bioinformatics* 19, 1412-1416
21. Mirkin, B.G., *et al.* (2003) Algorithms for computing parsimonious evolutionary scenarios for genome evolution, the last universal common ancestor and dominance of horizontal gene transfer in the evolution of prokaryotes. *BMC Evol Biol* 3, 2
22. Cline, M.S., *et al.* (2007) Integration of biological networks and gene expression data using Cytoscape. *Nat Protoc* 2, 2366-2382

23. Schiering, N., *et al.* (1995) Structures of the apo- and the metal ion-activated forms of the diphtheria tox repressor from Corynebacterium diphtheriae. *Proc Natl Acad Sci U S A* 92, 9843-9850
24. Cook, W.J., *et al.* (1998) Crystal structure of the cyanobacterial metallothionein repressor SmtB: a model for metalloregulatory proteins. *J Mol Biol* 275, 337-346
25. Alekshun, M.N., *et al.* (2001) The crystal structure of MarR, a regulator of multiple antibiotic resistance, at 2.3 A resolution. *Nat Struct Biol* 8, 710-714
26. Schreiter, E.R., *et al.* (2003) Crystal structure of the nickel-responsive transcription factor NikR. *Nat Struct Biol* 10, 794-799
27. Gury, J., *et al.* (2004) Cloning, deletion, and characterization of PadR, the transcriptional repressor of the phenolic acid decarboxylase-encoding padA gene of Lactobacillus plantarum. *Appl Environ Microbiol* 70, 2146-2153
28. Lee, S.J., *et al.* (2003) TrmB, a sugar-specific transcriptional regulator of the trehalose/maltose ABC transporter from the hyperthermophilic archaeon Thermococcus litoralis. *J Biol Chem* 278, 983-990
29. Schell, M.A. (1993) Molecular biology of the LysR family of transcriptional regulators. *Annu Rev Microbiol* 47, 597-626
30. Nguyen, C.C., and Saier, M.H., Jr. (1995) Phylogenetic, structural and functional analyses of the LacI-GalR family of bacterial transcription factors. *FEBS Lett* 377, 98-102
31. Hillerich, B., and Westpheling, J. (2006) A new GntR family transcriptional regulator in streptomyces coelicolor is required for morphogenesis and antibiotic production and controls transcription of an ABC transporter in response to carbon source. *J Bacteriol* 188, 7477-7487
32. Escolar, L., *et al.* (1998) Binding of the fur (ferric uptake regulator) repressor of Escherichia coli to arrays of the GATAAT sequence. *J Mol Biol* 283, 537-547
33. Campoy, S., *et al.* (2002) A new regulatory DNA motif of the gamma subclass Proteobacteria: identification of the LexA protein binding site of the plant pathogen Xylella fastidiosa. *Microbiology* 148, 3583-3597
34. Miller, M.B., and Bassler, B.L. (2001) Quorum sensing in bacteria. *Annu Rev Microbiol* 55, 165-199
35. Goldberg, M.D., *et al.* (2001) Role of the nucleoid-associated protein Fis in the regulation of virulence properties of enteropathogenic Escherichia coli. *Mol Microbiol* 41, 549-559
36. Kormanec, J., *et al.* (1998) The Streptomyces aureofaciens homologue of the whiB gene is essential for sporulation; its expression correlates with the developmental stage. *Folia Microbiol (Praha)* 43, 605-612
37. Guedon, E., *et al.* (2001) Pleiotropic transcriptional repressor CodY senses the intracellular pool of branched-chain amino acids in Lactococcus lactis. *Mol Microbiol* 40, 1227-1239
38. Nanamiya, H., *et al.* (2003) Involvement of ClpX protein in the post-transcriptional regulation of a competence specific transcription factor, ComK protein, of Bacillus subtilis. *J Biochem* 133, 295-302
39. Nair, S., *et al.* (2000) CtsR controls class III heat shock gene expression in the human pathogen Listeria monocytogenes. *Mol Microbiol* 35, 800-811
40. Zhao, H., *et al.* (2002) DNA complexed structure of the key transcription factor initiating development in sporulating bacteria. *Structure* 10, 1041-1050
41. Arnqvist, A., *et al.* (1992) The Crl protein activates cryptic genes for curli formation and fibronectin binding in Escherichia coli HB101. *Mol Microbiol* 6, 2443-2452
42. Martin, M., *et al.* (2000) MucR is necessary for galactoglucan production in Sinorhizobium meliloti EFB1. *Mol Plant Microbe Interact* 13, 129-135

43. Wu, W.F., *et al.* (1993) MetJ-mediated regulation of the Salmonella typhimurium metE and metR genes occurs through a common operator region. *FEMS Microbiol Lett* 108, 145-150
44. Pruss, B.M., *et al.* (2001) FlhD/FlhC-regulated promoters analyzed by gene array and lacZ gene fusions. *FEMS Microbiol Lett* 197, 91-97
45. Klug, A. (1999) Zinc finger peptides for the regulation of gene expression. *J Mol Biol* 293, 215-218
46. Laity, J.H., *et al.* (2001) Zinc finger proteins: new insights into structural and functional diversity. *Curr Opin Struct Biol* 11, 39-46
47. Littlewood, T.D., and Evan, G.I. (1995) Transcription factors 2: helix-loop-helix. *Protein Profile* 2, 621-702
48. Amoutzias, G.D., *et al.* (2007) One billion years of bZIP transcription factor evolution: conservation and change in dimerization and DNA-binding site specificity. *Mol Biol Evol* 24, 827-835
49. Gehring, W.J. (1992) The homeobox in perspective. *Trends Biochem Sci* 17, 277-280
50. Kisseleva, T., *et al.* (2002) Signaling through the JAK/STAT pathway, recent advances and future challenges. *Gene* 285, 1-24
51. Wilson, V., and Conlon, F.L. (2002) The T-box family. *Genome Biol* 3, REVIEWS3008
52. Sutcliffe, J.E., *et al.* (2003) Tumour suppressors--a fly's perspective. *Eur J Cancer* 39, 1355-1362
53. Miyamoto, M., *et al.* (1988) Regulated expression of a gene encoding a nuclear factor, IRF-1, that specifically binds to IFN-beta gene regulatory elements. *Cell* 54, 903-913
54. Akai, J., and Storey, K. (2003) Brain or brawn: how FGF signaling gives us both. *Cell* 115, 510-512
55. Facchini, L.M., and Penn, L.Z. (1998) The molecular role of Myc in growth and transformation: recent discoveries lead to new insights. *FASEB J* 12, 633-651
56. Kagoshima, H., *et al.* (1993) The Runt domain identifies a new family of heteromeric transcriptional regulators. *Trends Genet* 9, 338-341
57. Akiyama, Y., *et al.* (1996) The gcm-motif: a novel DNA-binding motif conserved in Drosophila and mammals. *Proc Natl Acad Sci U S A* 93, 14912-14916
58. Shelest, E. (2008) Transcription factors in fungi. *FEMS Microbiol Lett* 286, 145-151
59. Machado, A.K., *et al.* (1997) Thioredoxin reductase-dependent inhibition of MCB cell cycle box activity in Saccharomyces cerevisiae. *J Biol Chem* 272, 17045-17054
60. MacPherson, S., *et al.* (2006) A fungal family of transcriptional regulators: the zinc cluster proteins. *Microbiol Mol Biol Rev* 70, 583-604
61. Keller, G., *et al.* (2005) Independent metalloregulation of Ace1 and Mac1 in Saccharomyces cerevisiae. *Eukaryot Cell* 4, 1863-1871
62. Sengupta, P., and Cochran, B.H. (1991) MAT alpha 1 can mediate gene activation by a-mating factor. *Genes Dev* 5, 1924-1934
63. Wang, Q., and Szaniszlo, P.J. (2007) WdStuAp, an APSES transcription factor, is a regulator of yeast-hyphal transitions in Wangiella (Exophiala) dermatitidis. *Eukaryot Cell* 6, 1595-1605
64. Ecker, J.R. (1995) The ethylene signal transduction pathway in plants. *Science* 268, 667-675
65. Yamasaki, K., *et al.* (2004) A novel zinc-binding motif revealed by solution structures of DNA-binding domains of Arabidopsis SBP-family transcription factors. *J Mol Biol* 337, 49-63
66. Eulgem, T., *et al.* (2000) The WRKY superfamily of plant transcription factors. *Trends Plant Sci* 5, 199-206

67. King, N., *et al.* (2008) The genome of the choanoflagellate Monosiga brevicollis and the origin of metazoans. *Nature* 451, 783-788
68. Pincus, D., *et al.* (2008) Evolution of the phospho-tyrosine signaling machinery in premetazoan lineages. *Proc Natl Acad Sci U S A* 105, 9680-9684
69. Manning, G., *et al.* (2008) The protist, Monosiga brevicollis, has a tyrosine kinase signaling network more elaborate and diverse than found in any known metazoan. *Proc Natl Acad Sci U S A* 105, 9674-9679
70. Carroll, K., *et al.* (2004) Tubby proteins: the plot thickens. *Nat Rev Mol Cell Biol* 5, 55-63
71. Wu, C.L., *et al.* (1995) In vivo association of E2F and DP family proteins. *Mol Cell Biol* 15, 2536-2546
72. Jones, P.G., *et al.* (1996) Cold shock induces a major ribosomal-associated protein that unwinds double-stranded RNA in Escherichia coli. *Proc Natl Acad Sci U S A* 93, 76-80
73. Riano-Pachon, D.M., *et al.* (2008) Green transcription factors: a chlamydomonas overview. *Genetics* 179, 31-39
74. Souer, E., *et al.* (1996) The no apical meristem gene of Petunia is required for pattern formation in embryos and flowers and is expressed at meristem and primordia boundaries. *Cell* 85, 159-170
75. Pellegrini, L., *et al.* (1995) Structure of serum response factor core bound to DNA. *Nature* 376, 490-498
76. Bowman, J.L., *et al.* (2002) Establishment of polarity in angiosperm lateral organs. *Trends Genet* 18, 134-141
77. Chang, C., and Shockey, J.A. (1999) The ethylene-response pathway: signal perception to gene regulation. *Curr Opin Plant Biol* 2, 352-358
78. Navaud, O., *et al.* (2007) TCP transcription factors predate the emergence of land plants. *J Mol Evol* 65, 23-33
